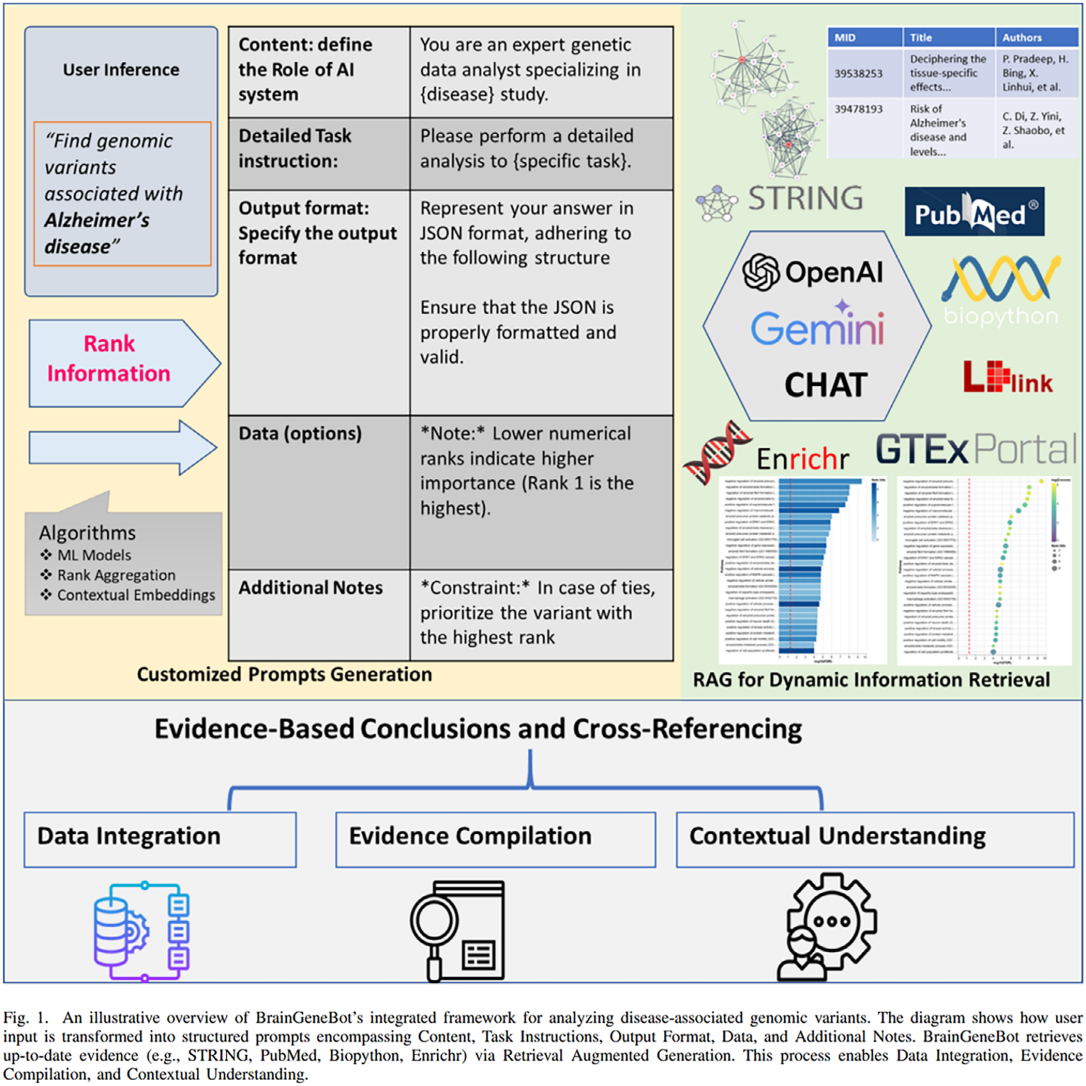# BrainGeneBot: A GPT‐engineered, user‐driven genetic data exploration with polygenic risk scores ranking in Alzheimer's disease

**DOI:** 10.1002/alz70855_107413

**Published:** 2025-12-25

**Authors:** Gang Qu, Zhongming Zhao

**Affiliations:** ^1^ University of Texas Health Science Center at Houston, Houston, TX, USA; ^2^ School of Biomedical Informatics, The University of Texas Health Science Center at Houston, Houston, TX, USA

## Abstract

**Background:**

Polygenic risk scores (PGSs) aggregate the effects of multiple genetic variants to attribute to disease susceptibility, and have been applied to numerous complex diseases including Alzheimer's disease (AD). However, as omics and phenotypic data grow exponentially, reconciling findings across studies with differing ancestral backgrounds, study designs, and statistical methodologies remains a pressing issue. Efficiently extracting biologically relevant insights from this expanding body of data is equally challenging, especially for diseases driven by complex interactions among genetic, environmental, and lifestyle factors.

**Method:**

To address these challenges, we introduce **BrainGeneBot**, an AI‐driven chatbot framework that automates genetic data analysis and bridges the gap between data generation and knowledge discovery. BrainGeneBot leverages a GPT‐based bot to facilitate user‐driven queries, advanced rank aggregation algorithms to reconcile varied datasets, and supervised learning in a transductive framework to prioritize genetic variants. It also integrates additional functionalities—such as protein interaction network construction, gene set enrichment, and literature retrieval from PubMed and NCBI—creating a unified platform for harmonizing, interpreting, and visualizing genomics data. This approach aims to streamline cross‐study comparisons and ensure reproducible outcomes.

**Result:**

By unifying variant rankings under conditions of low or zero overlap, it addresses a key challenge of rank aggregation algorithms in heterogenous datasets. BrainGeneBot's capabilities enable consensus prioritization of genetic variants while seamlessly linking them to biological pathways, thus offering a comprehensive tool for variant interpretation and discovery. This approach allows researchers to move beyond population‐level risk estimates, deriving biological insights from diverse genomic data sources and fostering a more nuanced understanding of complex diseases.

**Conclusion:**

The implementation of BrainGeneBot signifies a leap forward in advancing AD and neurodegenerative research by fostering data accessibility and accelerating discovery. Through AI‐driven automation and robust analytics, it refines the precision of genetic insights, translating statistical measures into meaningful biological interpretations. This integrative framework not only enhances knowledge discovery and hypothesis generation, but also ensures that findings remain actionable and reproducible, ultimately pushing the boundaries of genomic research for AD and potentially other brain disorders.